# Cryogel‐Based Dendritic Cell Immunotherapy for Post‐Surgical Breast Cancer Treatment

**DOI:** 10.1002/advs.202503238

**Published:** 2025-07-11

**Authors:** Lam‐Duc‐Huy Nguyen, Sheng‐Liang Cheng, Yu‐Ting Yen, Hsin‐Mei Lee, Te‐Haw Wu, Jane Wang, Shu‐Yi Lin, Yunching Chen

**Affiliations:** ^1^ Institute of Biomedical Engineering National Tsing Hua University Hsinchu 30013 Taiwan; ^2^ Department of Chemical Engineering National Tsing Hua University Hsinchu 34881 Taiwan; ^3^ International Intercollegiate PhD Program National Tsing Hua University Hsinchu 30013 Taiwan; ^4^ Institute of Translational Medicine and New Drug Development School of Medicine China Medical University Taichung 38019 Taiwan; ^5^ Institute of Biomedical Engineering and Nanomedicine National Health Research Institutes Miaoli 35053 Taiwan; ^6^ Department of Chemistry National Tsing Hua University Hsinchu 30013 Taiwan

**Keywords:** cryogel, dendritic cell vaccine, immunotherapy, triple‐negative breast cancer

## Abstract

Triple‐negative breast cancer (TNBC) is an aggressive malignancy with high mortality and limited treatment options. While surgical resection removes the primary tumor, it often fails to prevent recurrence or metastasis, and despite the promise of immunotherapy, response to immune checkpoint blockade remains poor. Here, a cryogel‐based dendritic cell (DC) immunotherapy is developed incorporating gold nanodot‐lipopolysaccharide (AuLPS)‐loaded DCs, doxorubicin (Dox), and PD‐1 immune checkpoint blockade (aPD‐1+Dox+AuLPS@DC) to enhance post‐surgical antitumor immunity. The AuLPS nanoparticles (NPs) stabilize LPS assembly, optimizing Th1 adjuvant activity and improving DC immunotherapy efficacy while minimizing adverse effects. The cryogel enables the sustained, localized release of therapeutic agents at the surgical site, preserving DC viability, migration, and functionality within the tumor microenvironment. This strategy enhances DC activation and potentiates robust T‐cell activation in both tumor‐draining lymph nodes and tumor beds, leading to durable antitumor immunity. When administered at the post‐surgical site in an orthotopic TNBC model, the aPD‐1+Dox+AuLPS@DC cryogel immunotherapy significantly delays tumor recurrence, reduces distant metastasis, and prolongs survival. These findings highlight cryogel‐based DC immunotherapy as a promising post‐surgical therapeutic strategy to enhance responses to immune checkpoint blockade and improve outcomes in TNBC.

## Graphical abstract

A multifunctional GelMA cryogel enables dendritic cell immunotherapy that synergizes with doxorubicin and PD‐1 blockade, driving robust post‐surgical T‐cell immunity and suppressing TNBC recurrence.

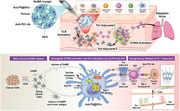

## 1 Introduction

Triple‐negative breast cancer (TNBC) is an aggressive malignancy with high mortality and limited treatment options, making it one of the most challenging therapeutic frontiers in oncology.^[^ ^1^ ^]^ While surgical resection remains a cornerstone of treatment, residual cancer cells can persist post‐surgery, leading to tumor recurrence and metastasis.^[^ ^2^, ^3^ ^]^ In recent years, immunotherapy has emerged as a transformative approach by leveraging the host immune system to combat malignancies.^[^ ^4^, ^5^ ^]^ Notably, when used in a neoadjuvant or adjuvant setting alongside surgery, immunotherapy has demonstrated long‐term benefits for TNBC patients.^[^ ^6^, ^7^, ^8^, ^9^ ^]^

Among various immunotherapeutic strategies, dendritic cell (DC)‐based vaccines have obtained substantial attention. As key antigen‐presenting cells (APCs), DCs play a central role in stimulating tumor‐specific effector T cells to suppress tumor growth while also eliciting long‐term immunological memory to prevent recurrence.^[^ ^10^, ^11^, ^12^, ^13^, ^14^ ^]^ However, the clinical translation of DC vaccines remains limited. A significant portion of administered DCs undergo rapid apoptosis, with only a small fraction successfully reaching tumor‐draining lymph nodes.^[^ ^15^ ^]^ Moreover, even when properly localized, DCs may fail to activate effectively or present antigens efficiently, which is crucial for inducing a robust immune response.^[^ ^16^, ^17^ ^]^ Additionally, the immunosuppressive tumor microenvironment (TME) can further diminish DC vaccine efficacy through mechanisms such as downregulating immunogenic epitopes or expressing ligands that inhibit effector T‐cell activity.^[^ ^18^, ^19^ ^]^ Thus, there is a critical need for strategies that enhance DC survival, promote their migration to lymphoid tissues, and potentiate T cell‐driven antitumor immunity.

Given these challenges, adjuvants play a crucial role in improving DC vaccine efficacy by enhancing both DC activation and antigen presentation.^[^ ^20^, ^21^ ^]^ However, conventional adjuvants can be a double‐edged sword—while they may amplify immune responses, they can also trigger excessive inflammation, leading to systemic toxicity and reduced therapeutic efficacy.^[^ ^22^ ^]^ As a result, research efforts have focused on developing innovative adjuvants that synergize with DC vaccines to mount a more precise and effective immune response. One promising approach involves the controlled manipulation of lipopolysaccharide (LPS) aggregates.^[^ ^23^, ^24^ ^]^ LPS, a key bacterial component composed of an O antigen, a core carbohydrate, and a lipid A moiety, induces diverse inflammatory responses depending on its aggregate structure. Transitioning LPS aggregates from lamellar to non‐lamellar phases (e.g., cubosomes and hexosomes) can trigger a potent immune cascade, but excessive immune activation can also lead to severe cytokine storms and sepsis.^[^ ^25^ ^]^ Building on our previous study, we developed gold nanodot‐stabilized LPS nanoparticles (AuLPS NPs) to modulate LPS assembly and balance its immunostimulatory effects. This design maintains a stable equilibrium between lamellar and non‐lamellar LPS phases, thereby optimizing Th1 adjuvant activity without excessive toxicity.^[^ ^26^ ^]^ We hypothesize that incorporating AuLPS NPs into DCs will polarize them toward a Th1‐mediated immune response, ultimately enhancing the efficacy of DC‐based immunotherapy.

Beyond molecular strategies, ensuring DC survival, prolonging their functionality, and achieving sustained immunotherapeutic release necessitate innovative delivery platforms.^[^ ^27^ ^]^ To address this, we formulated a cryogel‐based DC immunotherapy using a gelatin methacryloyl (GelMA)‐based macroporous cryogel scaffold. Cryogels are fabricated by crosslinking at subzero temperatures (‐5 to ‐20 °C), forming a highly porous (≈100 µm pore size) yet mechanically stable network upon thawing. This structure provides a 3D microenvironment conducive to cell viability, migration, and interaction while enabling co‐loading of multiple therapeutic agents.^[^ ^28^ ^]^ Unlike conventional hydrogels, cryogels feature interconnected macropores that enhance cellular activity and sustain the functionality of encapsulated DCs.^[^ ^29^, ^30^, ^31^, ^32^ ^]^

**Figure 1:**
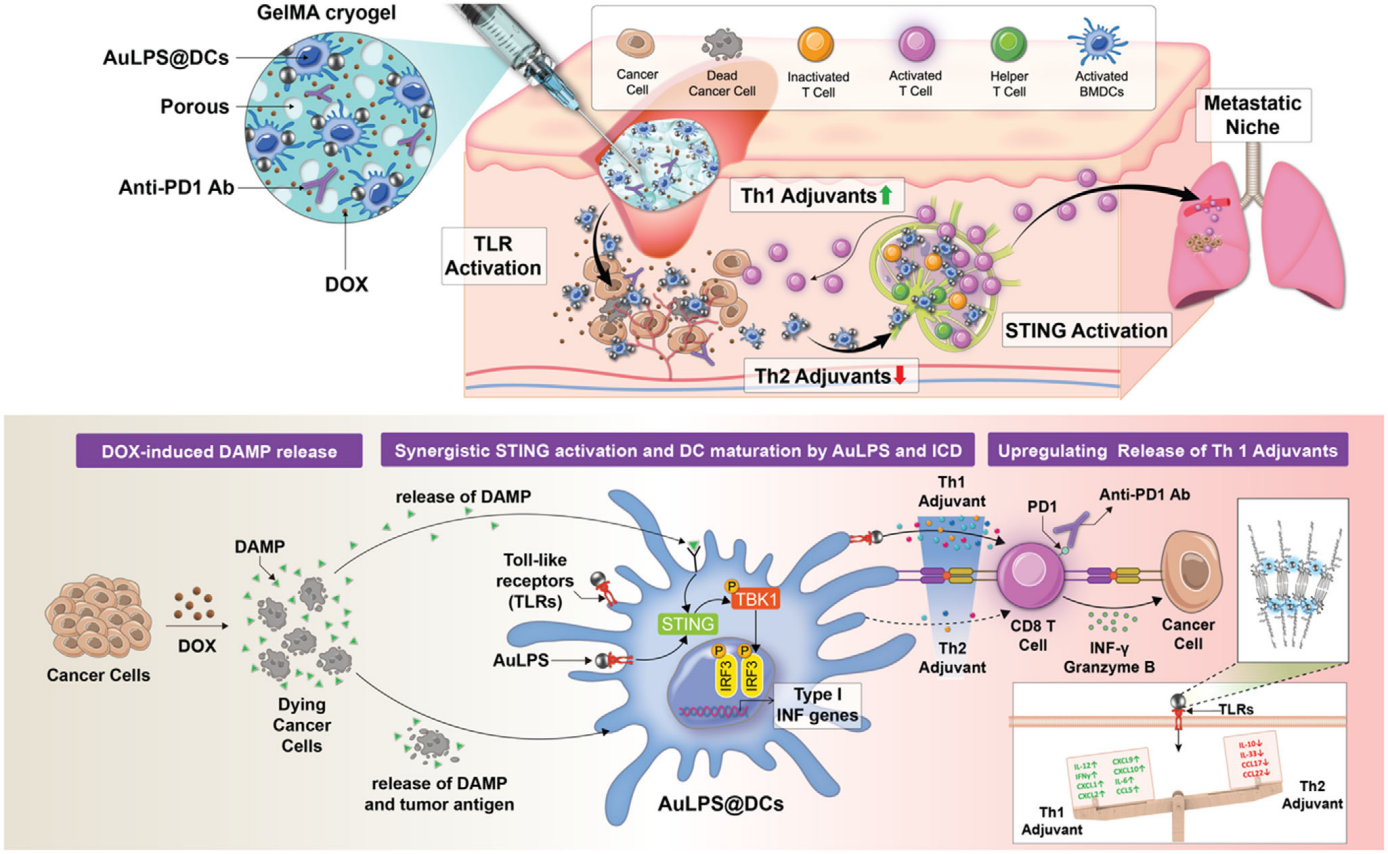
Schematic representation of the GelMA cryogel‐derived dendritic cell immunotherapy architecture and the mechanisms of antitumor immunity induction for post‐surgical breast cancer treatment. We demonstrate that a dendritic cell (DC) immunotherapy derived from GelMA cryogel, which delivers gold nanodot‐lipopolysaccharide (AuLPS)‐loaded DCs, the chemotherapeutic agent doxorubicin, and a PD‐1 immune checkpoint blockade, effectively induces immune cell infiltration and activates both DCs and cytotoxic T cells in tumor beds and tumor‐draining lymph nodes, resulting in a strong anticancer response. The cryogels facilitate the sustained release of these therapeutic agents at surgical sites, maintaining the viability and functionality of DCs within the tumor microenvironment. Post‐surgical injection into tumor sites enables doxorubicin to target and eliminate tumor cells, leading to immunogenic cell death (ICD). Tumor antigens released from the dead cancer cells are captured by DCs within the cryogel, promoting their maturation and migration to the draining lymph nodes. The AuLPS nanoparticle (NP) stabilizes LPS assembly, optimizing Th1 adjuvant activity and thereby enhancing the efficacy of DC‐based immunotherapy while reducing adverse effects. This process leads to the activation and expansion of cytotoxic T cells, contributing to the suppression of tumor regrowth post‐surgery, inhibition of distant metastasis, and improved survival outcomes. Moreover, incorporating an immune checkpoint blockade (anti‐PD‐1 antibody) into the GelMA cryogel is designed to counteract the immunosuppressive nature of the tumor microenvironment, further enhancing the overall immune response.

Within this cryogel scaffold, we encapsulated AuLPS NP‐loaded DCs, the chemotherapeutic agent doxorubicin (Dox), and an immune checkpoint blockade (anti‐PD‐1 antibody). **Figure** **1** illustrates the design and therapeutic advantages of this GelMA cryogel‐based DC immunotherapy. Upon administration at the post‐surgical tumor site, Dox induces immunogenic cell death (ICD), releasing tumor antigens that are subsequently captured by AuLPS NP‐loaded DCs within the cryogel. This enables local antigen sensing and provides in situ DC vaccine‐like properties without the need for ex vivo antigen loading. These DCs mature and migrate to the draining lymph nodes, where they prime cytotoxic T cells, leading to a robust antitumor immune response. The activated CD8^+^ T cells then suppress tumor recurrence and inhibit distant metastasis. Furthermore, integrating PD‐1 blockade into the GelMA cryogel helps counteract TME‐induced immunosuppression, thereby enhancing overall therapeutic efficacy. This approach offers a promising post‐surgical immunotherapy strategy that improves DC activity, enhances antitumor immunity, and addresses the limitations of conventional immune checkpoint blockade in TNBC treatment.

## 2 Results and Discussion

### 2.1 Engineering AuLPS NPs for Enhanced Th1 Dendritic Cell Activation

**Figure 2:**
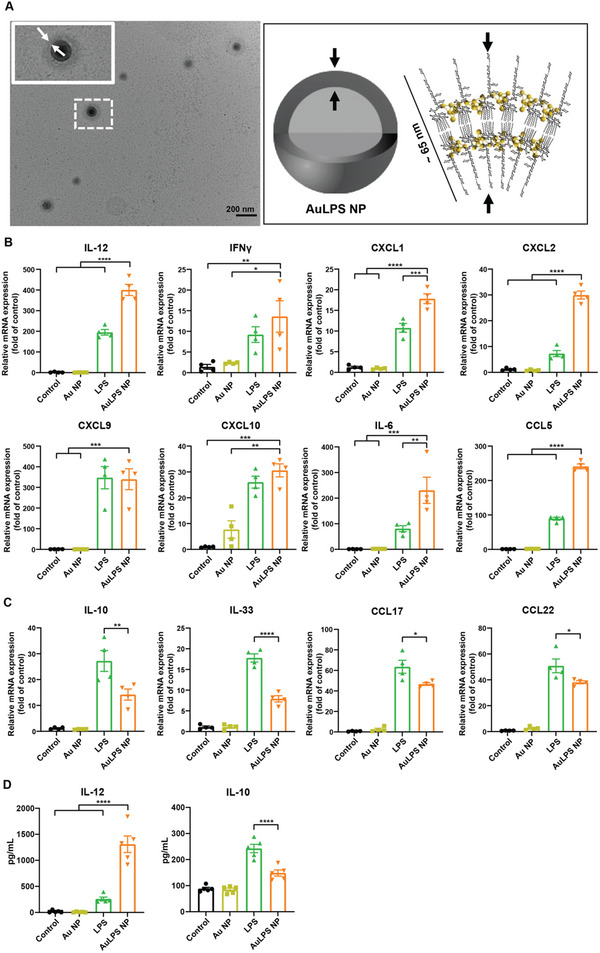
Gold nanodot‐lipopolysaccharide (AuLPS) nanoparticles (NPs) enhance the characteristics of Th1 adjuvants and reduce the characteristics of Th2 adjuvants in DCs. A) Schematic illustration of AuLPS nanoparticle (NP) assembly, formed by co‐assembling hydrophilic gold nanodots (AuNDs) with lipopolysaccharides (LPS). Transmission electron microscopy (TEM) images confirm their spherical, vesicle‐like morphology with a bilayer structure. Scale bar, 200 nm. B,C) The mRNA expression levels of Th1 characteristic adjuvants and inflammation‐related genes (*Il12, Ifng*, *Cxcl1, Cxcl2, Cxcl9, Cxcl10, Il6, and Ccl5*) (B) and Th2 characteristic adjuvants and anti‐inflammation‐related genes (*Il10, il33, Ccl17, and Ccl22*) (C) in BMDCs 24 h after treatment with free LPS, gold nanodot (Au) NPs, or AuLPS NPs were measured by RT–qPCR. The results are expressed as fold changes relative to the corresponding level in the untreated control group (*n* = 4). D) Cytokine secretion from BMDCs was measured by ELISA 12 h after treatment with free LPS, Au NPs or AuLPS NPs (*n* = 5). LPS: 0.1 µg mL^−1^; gold nanodot (Au) NPs: 0.1 mg mL^−1^. All data are shown as the mean ± SEM. *p < 0.05, ^**^ *p* < 0.01, ^***^ *p* < 0.001, ^****^ *p* < 0.0001.

The AuLPS NPs were prepared by co‐assembling hydrophilic gold nanodots, synthesized using G4NH2 dendrimers, with LPS. The G4NH2 dendrimers with gold nanodots preserve a hydrophilic surface that facilitates the controlled assembly of LPS molecules into stable nanovesicles, suppressing the formation of highly active cubic and hexagonal phases. The AuLPS NPs exhibited an average size exceeding 200 nm, as determined by dynamic light scattering (DLS), with transmission electron microscopy (TEM) confirming their spherical morphology and uniform vesicle‐like structure (**Figure** **2**A). Within the nanovesicles, LPS molecules adopted a tail‐to‐tail arrangement, forming a bilayer with a wall thickness of ≈65 nm, indicating dense lipid A packing (Figure 2A). This configuration, enabled by the hydrophilic properties of the G4NH2‐based AuNDs, enhances the immunogenic potential of LPS while ensuring stability and controlled activation, making AuLPS NPs a promising platform for modulating immune responses.

To evaluate their potential, we investigated the ability of AuLPS NPs to enhance Th1 immune responses in DCs, a key target of cancer immunotherapy. The levels of Th1‐associated and pro‐inflammatory cytokines were determined in bone marrow‐derived dendritic cells (BMDCs) after loading AuLPS NPs, using real‐time reverse transcription‐polymerase chain reaction (RT‐PCR). Compared to the Au NPs and the untreated control, AuLPS NPs generated a stronger Th1 immune response, as indicated by increased expression of Th1‐associated cytokines (IL‐12 and IFN‐γ), innate chemotactic chemokines (CXCL1, CXCL2, CXCL9, and CXCL10), and other inflammation‐related genes (IL‐6 and CCL5) (Figure 2B). LPS moderately upregulated Th1‐associated and pro‐inflammatory cytokines. In contrast, loading AuLPS NPs in DCs induced a weaker Th2 immune response, as indicated by a moderate increase in Th2‐associated cytokines (IL‐10, IL‐33, CCL17, and CCL22) compared to LPS treatment (Figure 2C). Consistent with mRNA expression, AuLPS NPs significantly increased Th1‐associated cytokine secretion (IL‐12) and reduced Th2‐associated cytokine secretion (IL‐10) compared to LPS in free form (Figure 2D; Figure S1, Supporting Information). These results indicate the potential of AuLPS NPs in enhancing Th1 immune responses, which are crucial for robust antitumor immunity. By significantly upregulating Th1‐associated cytokines and pro‐inflammatory markers, AuLPS NPs demonstrate strong potential as adjuvants for enhancing Th1‐type immune responses. The increased Th1/Th2 ratio induced by AuLPS NPs further highlights the shift toward a Th1‐dominated immune response, which is favorable for antitumor activity.

### 2.2 Synergistic Activation of STING Signaling by AuLPS NPs and Chemotherapy for Enhanced Dendritic Cell Maturation In Vitro

**Figure 3:**
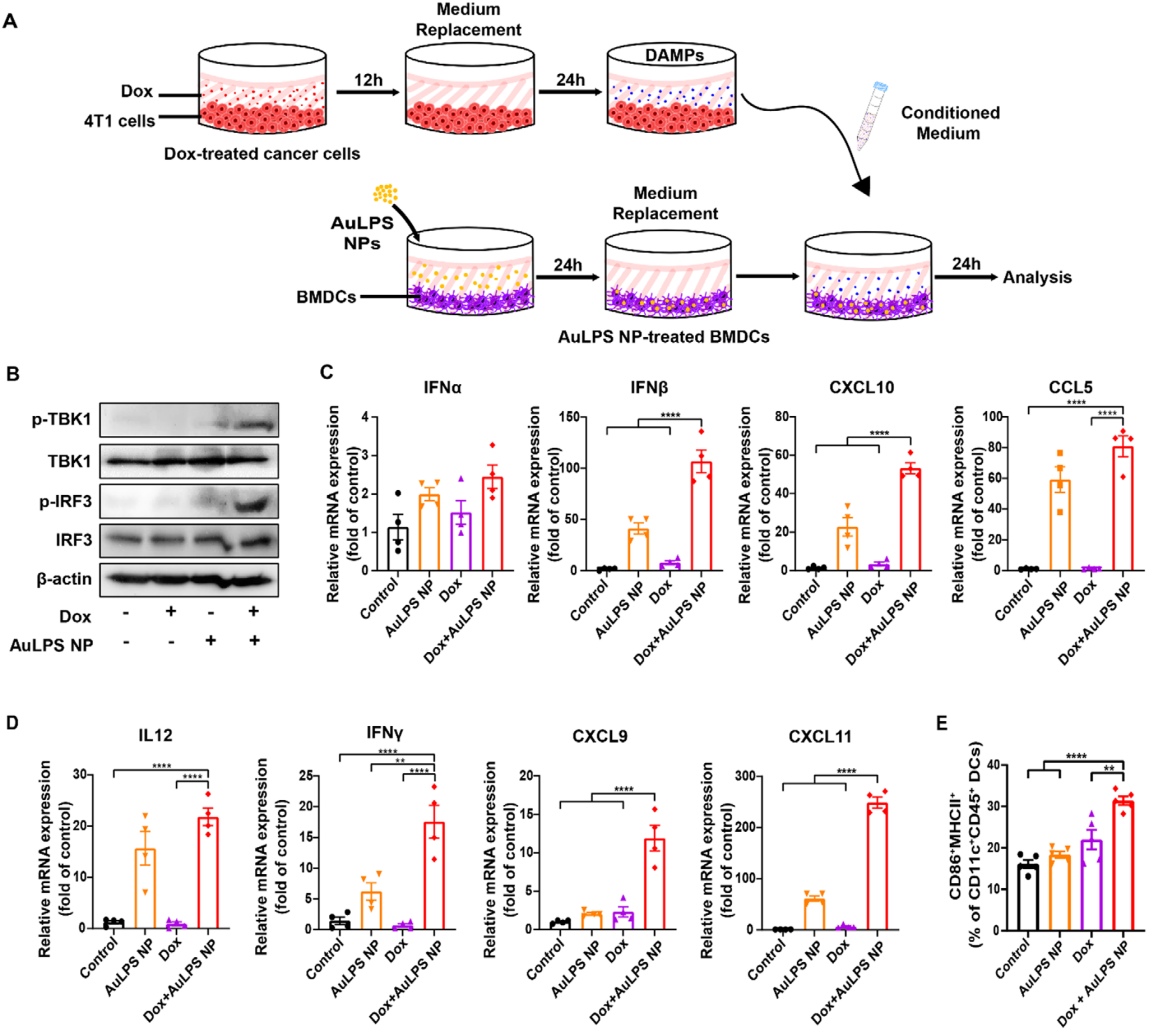
Enhanced STING activation and pro‐inflammatory cytokine production in BMDCs treated with AuLPS NPs and conditioned medium from Dox‐treated 4T1 cells. A) Schematic representation of the experimental setup. 4T1 cancer cells were treated with doxorubicin (Dox) for 12 h to induce immunogenic cell death (ICD). After 12 h, the medium was replaced with fresh medium and incubated for an additional 24 h to allow the release of damage‐associated molecular patterns (DAMPs). The resulting conditioned medium was collected and used to mimic the tumor microenvironment post‐chemotherapy. BMDCs were pretreated with AuLPS nanoparticles (NPs) for 24 h, followed by medium replacement with conditioned medium from Dox‐treated 4T1 cells for another 24 h to assess synergistic activation. B) Western blot analysis of TBK1 and IRF3 phosphorylation in BMDCs treated with conditioned medium from Dox‐treated 4T1 cells (Dox), AuLPS NPs, or a combination of both for 24 h. C,D) mRNA expression levels of type I IFNs (*Ifna1, Ifnb1, Cxcl10, Ccl5*) (C) and inflammation‐related genes (*Il12, Ifng, Cxcl9, Cxcl11*) (D) in BMDCs 24 h after treatment with conditioned medium from Dox‐treated 4T1 cells (Dox), AuLPS NPs, or a combination of both were measured by RT–qPCR. The results are expressed as fold changes relative to the corresponding levels in the untreated control group (*n* = 4). E) Flow cytometry analysis of MHC II and CD86 expression in BMDCs treated with conditioned medium from Dox‐treated 4T1 cells (Dox), AuLPS NPs, or a combination of both for 24 h (*n* = 5). LPS: 0.1 µg mL^−1^; gold nanodot (Au) NPs: 0.1 mg mL^−1^; Dox: 1 µm. All data are presented as the mean ± SEM. ^**^ *p* < 0.01, ^****^ *p* < 0.0001.

Numerous studies have demonstrated that combining DC‐based vaccines with chemotherapy, known as chemo‐immunotherapy, significantly enhances anticancer effects. The dying tumor cells release tumor‐derived DNA, which activates STING signaling in DCs to trigger immune responses, enhance antigen presentation, and stimulate cytotoxic T cells, thereby providing potent DC‐based cancer vaccines. To determine whether chemotherapy‐treated cancer cells could synergize with AuLPS NPs to activate DCs, we first examined the impact of conditioned medium from Dox‐treated 4T1 cells on the STING‐mediated immune response in BMDCs, with or without loading AuLPS NPs (**Figure** **3**A).

Exposure to both AuLPS NPs and conditioned medium from Dox‐treated 4T1 cells profoundly activated STING signaling, as evidenced by greater TBK1 and IRF3 activation (Figure 3B). Neither AuLPS NPs nor conditioned medium from Dox‐treated 4T1 cells alone significantly enhanced TBK1 and IRF3 phosphorylation in BMDCs (Figure 3B). As a result, exposure to AuLPS NPs and conditioned medium from Dox‐treated 4T1 cells significantly increased the gene expression of type I IFN‐related genes (Ifna1, Ifnb1, Cxcl10, and Ccl5) (Figure 3C) and pro‐inflammatory cytokines (Il12, Ifng, Cxcl9, and Cxcl11) (Figure 3D; Figure S2, Supporting Information), compared with AuLPS NPs or conditioned medium alone, leading to enhanced DC maturation (CD86^+^MHCII^+^) (Figure 3E; Figure S3, Supporting Information).

The synergy between AuLPS NPs and Dox‐treated tumor cell‐conditioned medium in activating STING signaling and enhancing DC maturation strongly supports their combined use in chemo‐immunotherapy. This combination potentiates the activation of type I IFN‐related genes and pro‐inflammatory cytokines, which are critical for effective antitumor immune responses. ICD‐inducing chemotherapy, such as Dox, causes the release of DAMPs and tumor‐derived DNA. Both can enhance the activation of the cGAS‐STING pathway, promoting the production of type I interferons and pro‐inflammatory cytokines, thereby enhancing DC maturation and stimulating cytotoxic T cells. Combining AuLPS NPs and Dox offers a promising strategy for developing potent DC‐based cancer immunotherapy.

### 2.3 Cryogel‐Based DC Immunotherapy Enhances DC Migration, Th1 Polarization, and T‐Cell‐Mediated Immune Gene Activation in Post‐Surgical TNBC

**Figure 4:**
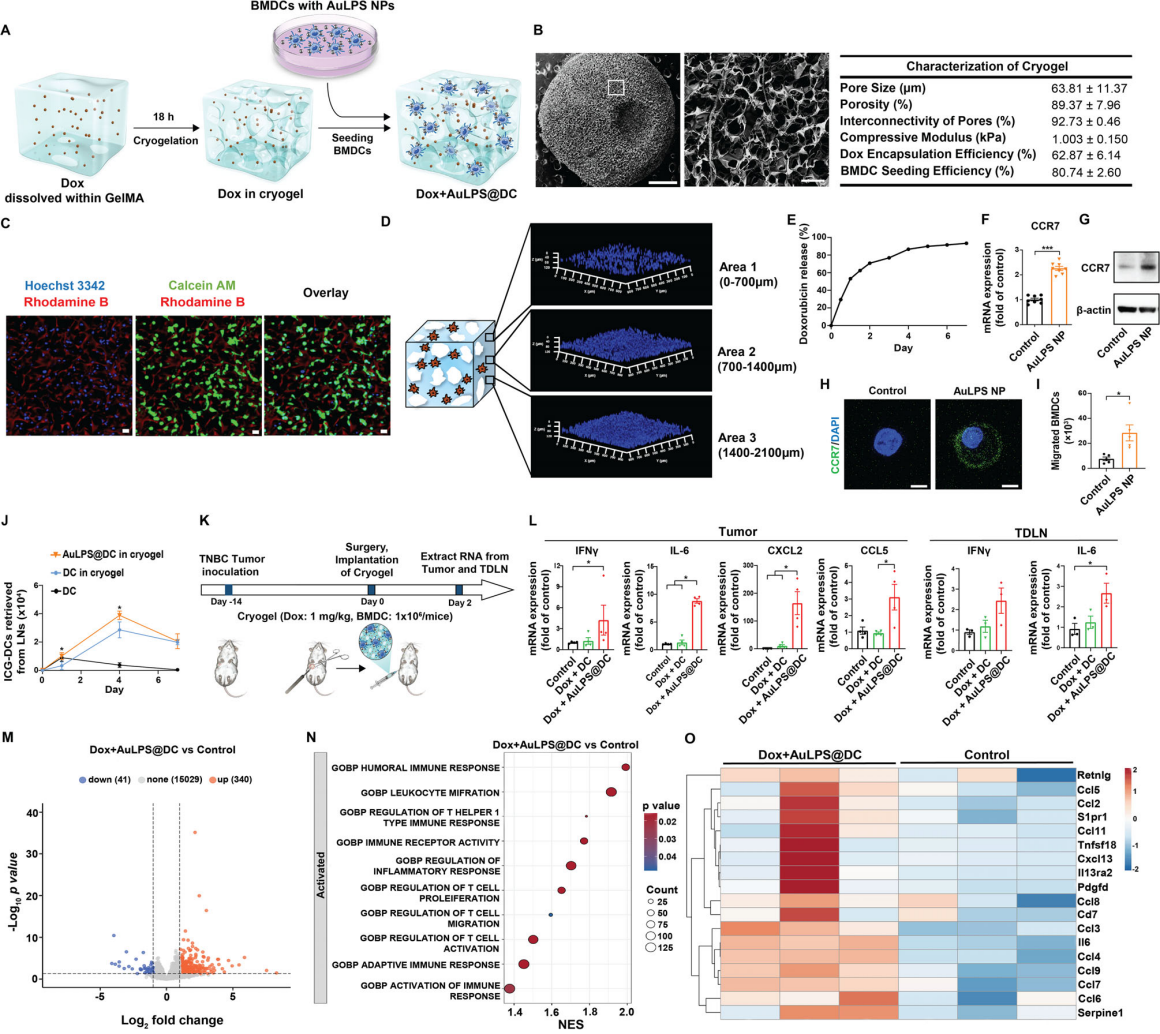
Cryogel‐based DC immunotherapy enables sustained Dox release, enhances DC viability and migration, and promotes immune activation. A) Synthesis of Dox+AuLPS@DC. Doxorubicin was incorporated into a GelMA (0.75% w/v) prepolymer solution before cryogelation at ‐20 °C for 18 h, forming macroporous cryogels. Bone marrow‐derived dendritic cells (BMDCs) were seeded onto the cryogel for 2 h, either preloaded with AuLPS NPs or left untreated. B) SEM image of the GelMA cryogel. Scale bars: 1 mm (left) and 100 µm (right). Pore size (*n* = 100 from 4 cryogels), porosity (*n* = 4), interconnectivity of the porous structure (*n* = 4), compressive modulus (*n* = 6), doxorubicin encapsulation efficiency (EE) of the cryogel (*n* = 3), and BMDC seeding efficiency of the cryogel (*n* = 6). C,D) Viability and distribution of BMDCs in the cryogel visualized under 2D images (C, scale bars: 20 µm), and 3D images at different layers captured using CLSM (D). E) The release profile of doxorubicin (*n* = 4). F) The mRNA expression levels of Ccr7 in BMDCs 24 h after treatment with AuLPS NPs were measured by RT–qPCR. Results are expressed as fold changes relative to the corresponding levels in the untreated control group (*n* = 8). G,H) Western blot analysis (G) and confocal microscopy images (H) show CCR7 expression in BMDCs treated with AuLPS NPs for 24 h. Scale bars: 5 µm. I) The migratory capacity of BMDCs treated with AuLPS NPs in response to CCL21 was evaluated using a Transwell migration assay (*n* = 5). J) ICG+DCs retrieved from dLNs on days 0, 1, 4, and 7 after implanting ICG+AuLPS@DC in cryogels at the surgical site post‐tumor removal. Tumors were established on day‐14 by injecting 4T1 cells into BALB/c mice, surgically removed on day 0, and treated with cryogels or free DCs (control). TDLNs were analyzed for ICG+DC migration via flow cytometry (*n* = 3). K) Schematic of the experimental protocol. On day ‐14, 1 × 10⁵ 4T1 cells were injected into the mammary gland of BALB/c mice. The implanted tumor was surgically removed on day 0, and various formulations were implanted into the surgical site (Dox: 1 mg kg^−1^, BMDC: 1 × 10⁶ cells mouse^−1^). Tumors and TDLNs were analyzed on day 2. L) The mRNA expression levels of Th1‐characteristic adjuvants and inflammation‐related genes (Ifng, Il6, Cxcl2, and Ccl5) in tumors or TDLNs 48 h after treatment with cryogels containing Dox and BMDCs or Dox and AuLPS@DCs were measured by RT–qPCR. Results are expressed as fold changes relative to the corresponding levels in the untreated control group (*n* = 4 in tumors and *n* = 3 in TDLNs). M) Volcano plot showing genes differentially expressed in TNBC tumors upon treatment with cryogels containing Dox and AuLPS@DCs (Dox+AuLPS@DC) compared with untreated controls (*n* = 3). N) Gene ontological analysis of differentially expressed genes related to immune activation pathways (*n* = 3). O) Heatmap showing the expression profiles of genes associated with T‐cell activation, proliferation, and migration in Dox+AuLPS@DC‐treated tumors compared with untreated controls (*n* = 3). All data are shown as the mean ± SEM. ^*^ *p* < 0.05, ^***^ *p* < 0.001.

To achieve the sustained release of DCs and immune‐stimulating agents at tumor sites, GelMA‐based macroporous cryogels with excellent biocompatibility were utilized to encapsulate AuLPS NPs‐loaded DCs and the chemotherapeutic agent doxorubicin (Dox) (**Figure** **4**A). The cryogel microstructure, as revealed by SEM, consisted of large interconnected macropores, with an average pore size of ≈60 µm (Figure 4B). The ability of cryogels to support the loading of BMDCs was examined. The BMDC‐seeding efficiency in the cryogels was around 80%. BMDCs were initially entrapped in the cryogels with optimal viability (Figure 4C; Figure S4, Supporting Information). 3D confocal reconstruction of BMDC‐seeded cryogels after 2 h of incubation showed efficient and homogeneous attachment of DCs within the gel pores (Figure 4D). In addition, doxorubicin, when dissolved within the GelMA prepolymer solution, became entrapped within the polymer matrix during the cryogelation process. The Dox‐loading efficiency of the cryogel was around 63% (Figure 4B). Continuous release of Dox from the cryogels occurred over 7 days, suggesting that the hydrogels achieved sustained release of small‐molecule chemotherapeutics (Figure 4E).

We further investigated whether AuLPS NPs could enhance BMDC migration to TDLNs, a critical process mediated by the CCR7/CCL21 axis.^[^ ^33^ ^]^ Our data showed a significant upregulation of CCR7 expression, both at the mRNA and protein levels, in BMDCs treated with AuLPS NPs (Figure 4F–H; Figure S5, Supporting Information). This upregulation of CCR7 suggests that AuLPS NPs play a key role in enhancing the migratory capacity of DCs. To further investigate this, we conducted an in vitro transwell migration assay where CCL21, the CCR7 ligand, was used as a chemoattractant. BMDCs, with or without AuLPS NP treatment, were placed in the upper chamber, and migration toward the CCL21 gradient in the lower chamber was quantified. The results indicated a significant increase in the migration of AuLPS NP‐treated BMDCs compared to untreated BMDCs, supporting the hypothesis that AuLPS NPs enhance CCR7‐mediated DC migration to TDLNs (Figure 4I).

Recent studies have demonstrated that immunotherapy in combination with surgery can enhance the long‐term prognosis of TNBC. Thus, we assessed the application of cryogel‐based DC immunotherapy combined with surgery in the 4T1 orthotopic TNBC model. In an orthotopic 4T1 TNBC model, we evaluated the migration of BMDCs following surgical resection of tumors. BMDCs were fluorescently labeled with indocyanine green (ICG) prior to administration and either loaded into cryogels with or without AuLPS NPs. Following tumor resection on day 14 post‐implantation, free BMDCs or BMDCs loaded in cryogels were applied at the surgical site. To minimize systemic exposure to free AuLPS NPs, the DCs were preincubated with AuLPS NPs ex vivo, followed by thorough washing to remove unbound nanoparticles before cryogel encapsulation. Migration of ICG‐labeled BMDCs to the TDLNs was then analyzed by flow cytometry at 24 h and 4 days post‐injection.

Free ICG‐labeled DCs showed rapid migration to the TDLNs at 24 h, peaking at that time point, but quickly declined to baseline by day 4 (Figure 4J). In contrast, cryogel‐loaded DCs, both with and without AuLPS NPs, showed sustained migration to the TDLNs, peaking at day 4 and remaining elevated at day 7 (Figure 4J). The migration of cryogel‐loaded BMDCs was significantly enhanced by the presence of AuLPS NPs, with CCR7 upregulation promoting more efficient migration to the TDLNs compared to BMDCs without AuLPS NPs (Figure 4J). Additionally, significantly higher activation levels of AuLPS NP‐loaded BMDCs, indicated by increased CD86 and MHCII expression, were observed within the cryogels, post‐surgical residual tumors, and TDLNs, further demonstrating that the AuLPS@DC successfully activates BMDCs (Figure S6, Supporting Information).

To test whether cryogels encapsulating Dox and AuLPS NP‐loaded DCs enhanced the production of immune‐stimulating cytokines in vivo, we applied cryogels containing Dox and BMDCs, with or without AuLPS NP loading, at the tumor resection site and measured the gene expression of immune‐stimulating cytokines in the remaining tumors and TDLNs 48 h after administration (Figure 4K). Consistent with the in vitro results, cryogels containing Dox and BMDCs loaded with AuLPS NPs (Dox+AuLPS@DC) significantly increased Th1‐associated cytokines (IFN‐γ) and pro‐inflammatory cytokines (IL‐6, CXCL2, and CCL5) in remaining tumor beds and TDLNs (Figure 4L), compared to cryogels containing Dox and unloaded BMDCs (Dox+DC), as well as the untreated control group. Transcriptome analysis of TNBC tumors 48 h post‐administration revealed extensive immune activation, with 340 upregulated genes (Figure 4M), particularly those involved in humoral immune responses, T‐helper 1 (Th1) immunity, and T‐cell receptor signaling (Figure 4N). Notably, genes associated with T‐cell activation, proliferation, and migration were significantly enriched, suggesting enhanced T‐cell‐mediated antitumor immunity (Figure 4O).

The integration of AuLPS NPs into DCs not only enhances DC migration and activation but also reshapes the TME by promoting robust T‐cell‐mediated immunity. These findings highlight the potential of Dox+AuLPS@DC cryogels as a promising strategy for post‐surgical TNBC immunotherapy.

### 2.4 Cryogel‐Based DC Immunotherapy Suppresses Tumor Recurrence and Metastasis by Enhancing DC and Cytotoxic T‐Cell Activation in Post‐Surgical TNBC

**Figure 5:**
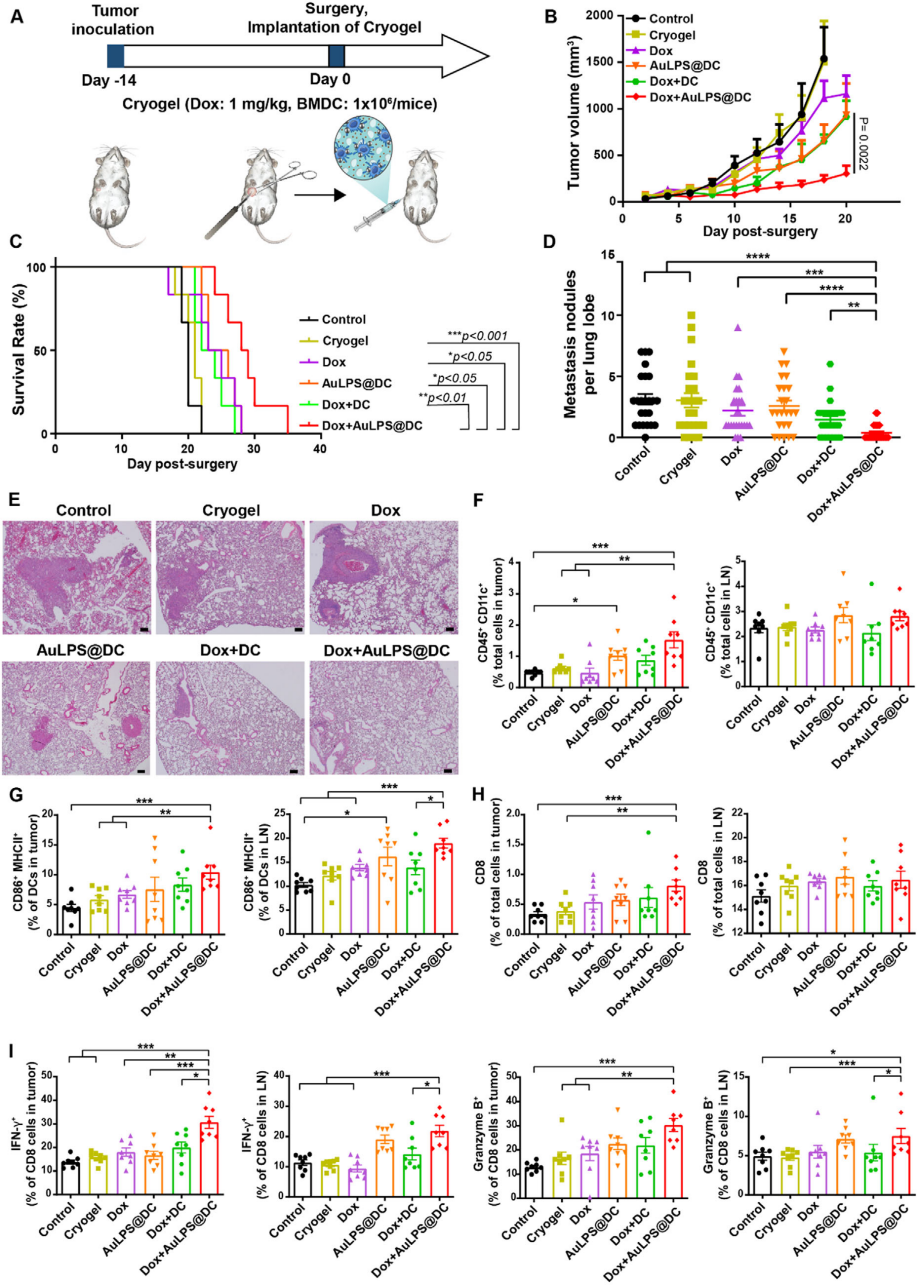
The cryogel containing AuLPS@DC and Dox inhibits postsurgical tumor recurrence in orthotopic TNBC and induces potent antitumor immunity. A) Schematic of the experimental protocol. On day ‐14, 1 × 10⁵ 4T1 cells were injected into the mammary gland of BALB/c mice. The implanted tumor was surgically removed on day 0, and various formulations were implanted into the surgical site (Dox: 1 mg kg^−1^, BMDC: 1 × 10⁶ cells mouse^−1^). Tumors and TDLNs were analyzed on day 14. B) The growth curve shows primary tumor regrowth after resection in the 4T1 TNBC model. C) Overall survival in the 4T1 TNBC model (*n* = 6). A comparison of survival curves was performed using the log‐rank Mantel–Cox test (two‐sided). D) Quantification of pulmonary metastatic nodules in mice on day 14 post‐surgery following treatment with various formulations (*n* = 24 lung lobes from 8 mice). E) Hematoxylin and eosin‐stained images showing metastatic tumor nodules in the lungs. Scale bars, 200 µm. F–I) Flow cytometry analysis of DCs (CD45⁺CD11c⁺) (F), activated DCs (CD86⁺MHCII⁺) (G), CD8⁺ T lymphocytes (CD3⁺CD8⁺) (H), and activated CD8⁺ T lymphocytes (IFN‐γ⁺ and granzyme B in CD8⁺) (I) in tumors and tumor‐draining lymph nodes (TDLNs) of mice (*n* = 8). All data are shown as the mean ± SEM. ^*^ *p* < 0.05, ^**^ *p* < 0.01, ^***^ *p* < 0.001.

We then evaluated whether cryogel‐based DC immunotherapy augmented the antitumor immune response in the 4T1 orthotopic TNBC model after the administration of different formulations at the surgical removal site (**Figure** **5**A). The cryogel encapsulating Dox and BMDCs loaded with AuLPS NPs (Dox+AuLPS@DC) significantly slowed tumor growth and improved overall survival in the clinically relevant post‐surgical TNBC model compared to the empty cryogel, Dox in cryogel (Dox), AuLPS@DC in cryogel (AuLPS@DC), or Dox and unloaded DC in cryogel (Dox+DC) (Figure 5B,C). Because mice bearing orthotopic 4T1 tumors develop metastases in the lungs, we further evaluated its effect on metastasis. We observed that Dox+AuLPS@DC in cryogel significantly suppressed distal lung metastasis in the TNBC model (Figure 5D,E). We then evaluated changes in TME in the 4T1 orthotopic TNBC model after the administration of different formulations at the surgical removal site. Our findings demonstrate that the cryogel encapsulating Dox and BMDCs loaded with AuLPS NPs (Dox+AuLPS@DC) significantly increased the proportion of DCs (CD45^+^CD11c^+^) in tumors (Figure 5F; Figure S7, Supporting Information) and the activation of DCs (CD86^+^MHCII^+^) in both tumors and TDLNs (Figure 5G; Figure S7, Supporting Information) compared to surgery alone. In addition, Dox+AuLPS@DC in cryogel moderately increased the proportion of cytotoxic T cells (CD3^+^CD8^+^) (Figure 5H; Figure S7, Supporting Information) in tumors and enhanced activation (CD8^+^IFN‐γ^+^ or CD8^+^Granzyme B^+^) (Figure 5I; Figure S7, Supporting Information) of cytotoxic T cells in both tumors and TDLNs. The number of CD3^+^CD4^+^ T cells in both tumors and TDLNs remained unchanged (Figure S8, Supporting Information).

**Figure 6:**
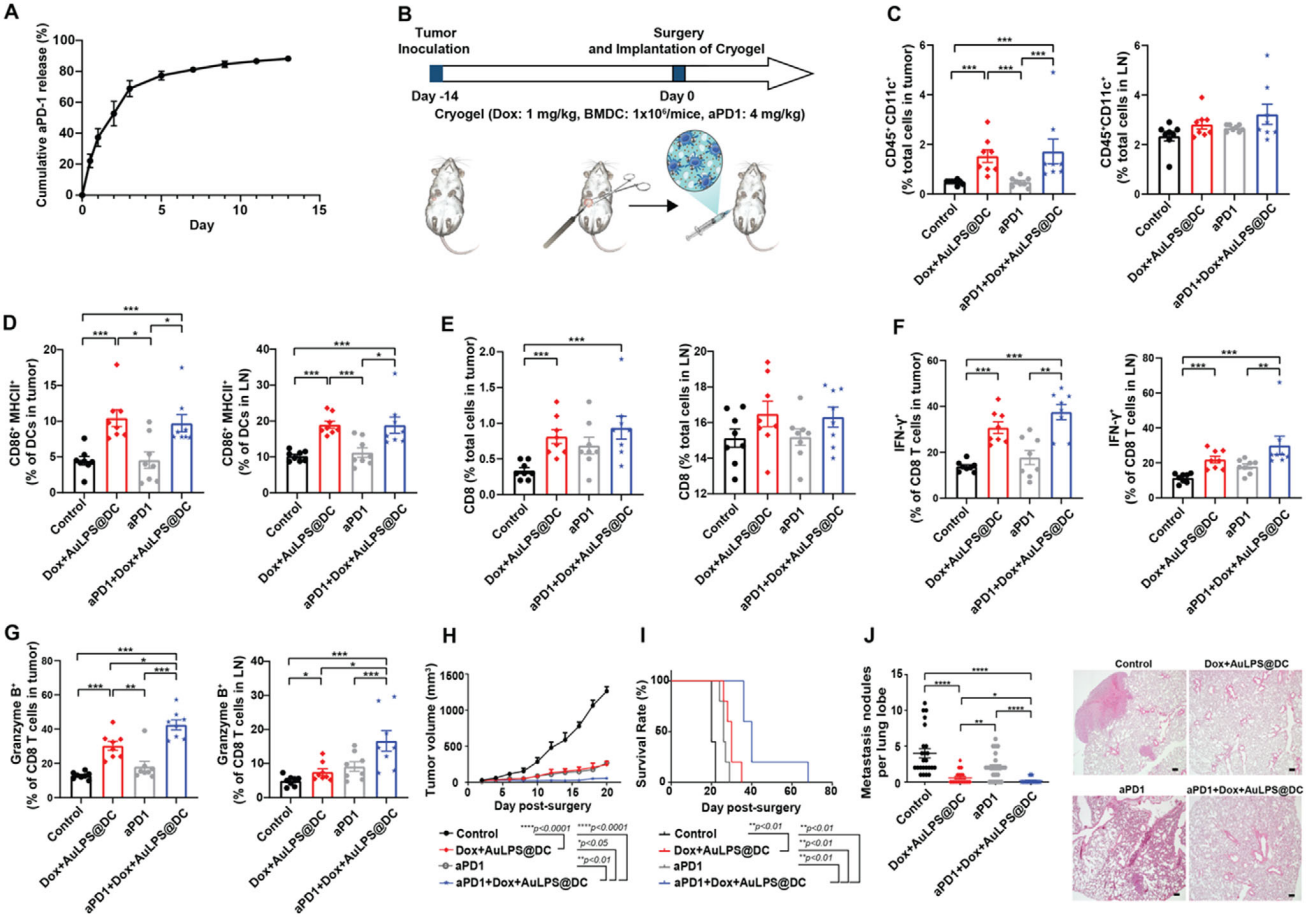
The cryogel containing AuLPS@DC, Dox, and an immunomodulator (anti‐PD‐1 antibody) shows the synergistic effect to achieve potent antitumor immunity and prevent postsurgical tumor recurrence in orthotopic TNBC. A) Release of Alexa Fluor 488 – anti‐PD‐1 from cryogel under physiological conditions (pH 7.4) (*n* = 3). B) Schematic of the experimental protocol. On day ‐14, 1 × 10^5^ 4T1 cells were injected into the mammary gland of BALB/c mice. The implanted tumor was surgically removed on day 0, and various formulations were implanted into the surgical site (Dox: 1 mg kg^−1^, BMDC: 1 × 10^6^ cells mouse^−1^, aPD1: 4 mg kg^−1^). Tumors and TDLNs were analyzed on day 14. (C‐G) DCs (CD45^+^CD11c^+^) C), activated DCs (CD86^+^MHCII^+^) D), CD8^+^ T lymphocytes (CD3^+^CD8^+^) E) and activated CD8^+^ T lymphocytes (IFN‐γ^+^ and granzyme B in CD8^+^) F,G) in tumor and tumor‐draining lymph nodes (TDLNs) of mice were detected by flow cytometry (*n* = 8). The data for the Control and Dox+AuLPS@DC groups presented in panels (C‐G) are shared with those shown in Figure 5F–I, as the experimental setups were identical. H) The growth curve shows the primary tumor re‐growth post‐resection in the 4T1 TNBC model. I) The overall survival in the 4T1 TNBC model (*n* = 5). A comparison of survival curves was performed using a log‐rank Mantel–Cox test (two‐sided). J) Quantification of pulmonary metastatic nodules in mice on day 14 post‐surgery following treatment with various formulations (*n*  =  24 lung lobes from 8 mice). Hematoxylin and eosin‐stained images showing metastatic tumour nodules in the lung. Scale bars, 200 µm. All data are shown as the mean ± SEM. ^*^ *p* < 0.05, ^**^ *p* < 0.01, ^***^ *p* < 0.001.

The PD‐1/PD‐L1 pathway, commonly exploited by tumors, dampens T‐cell activity and has become a key target in cancer immunotherapy. While PD‐1/PD‐L1 blockade can activate T‐cell function, its efficacy as monotherapy remains limited in TNBC. To overcome this limitation, we incorporated anti‐PD‐1 antibody alongside Dox and AuLPS@DC into the cryogel, aiming to reprogram the immunosuppressive TME and enhance checkpoint blockade efficacy. The cryogels successfully incorporated an immune checkpoint inhibitor, the anti‐PD‐1 antibody, achieving a high loading efficiency of 58.22 ± 4.19%. Similar to the results for Dox, we observed continuous release of anti‐PD‐1 antibody from the cryogels over a period of 7 days under physiological conditions (pH 7.4) (**Figure** **6**A).

We further investigated whether co‐encapsulation of the therapeutic anti‐PD‐1 antibody in the cryogels could synergize with Dox and AuLPS@DC in cryogels paired with surgery, aiming to generate a potent immune response and suppress cancer progression post‐tumor resection (Figure 6B). Our findings demonstrate that Dox+AuLPS@DC in cryogel with or without anti‐PD‐1 antibody in the cryogel significantly increased the proportion (CD45^+^CD11c^+^) (Figure 6C) of DCs in tumors and activation (CD86^+^MHCII^+^) (Figure 6D) of DCs in both tumors and TDLNs compared to surgery alone. Furthermore, when we incorporated the anti‐PD‐1 antibody into cryogel‐based DC immunotherapy (aPD1+Dox+AuLPS@DC in cryogel), it not only increased the proportion of CD3⁺CD8⁺ cytotoxic T cells within the tumor (Figure 6E) but also enhanced their activation, as evidenced by elevated expression of IFN‐γ and Granzyme B in both the tumors and TDLNs (Figure 6F,G). Our result highlights the potential of combining an anti‐PD‐1 antibody in cryogel‐based DC immunotherapy to enhance the antitumor immunity. As a result, aPD1+Dox+AuLPS@DC in cryogel significantly slowed tumor growth (Figure 6H), improved overall survival (Figure 6I), and suppressed distal lung metastasis (Figure 6J) than cryogel formulations containing either the anti‐PD‐1 antibody alone or the Dox+AuLPS@DC in cryogel. Collectively, our findings suggest that cryogel‐based DC immunotherapy, loaded with Dox, AuLPS@DC, and anti‐PD‐1 antibody, can stimulate robust antitumor immunity and suppress cancer recurrence in the postsurgical 4T1 TNBC model.

### 2.5 Systemic Safety Evaluation of Cryogel‐Based DC Immunotherapy In Vivo

**Figure 7:**
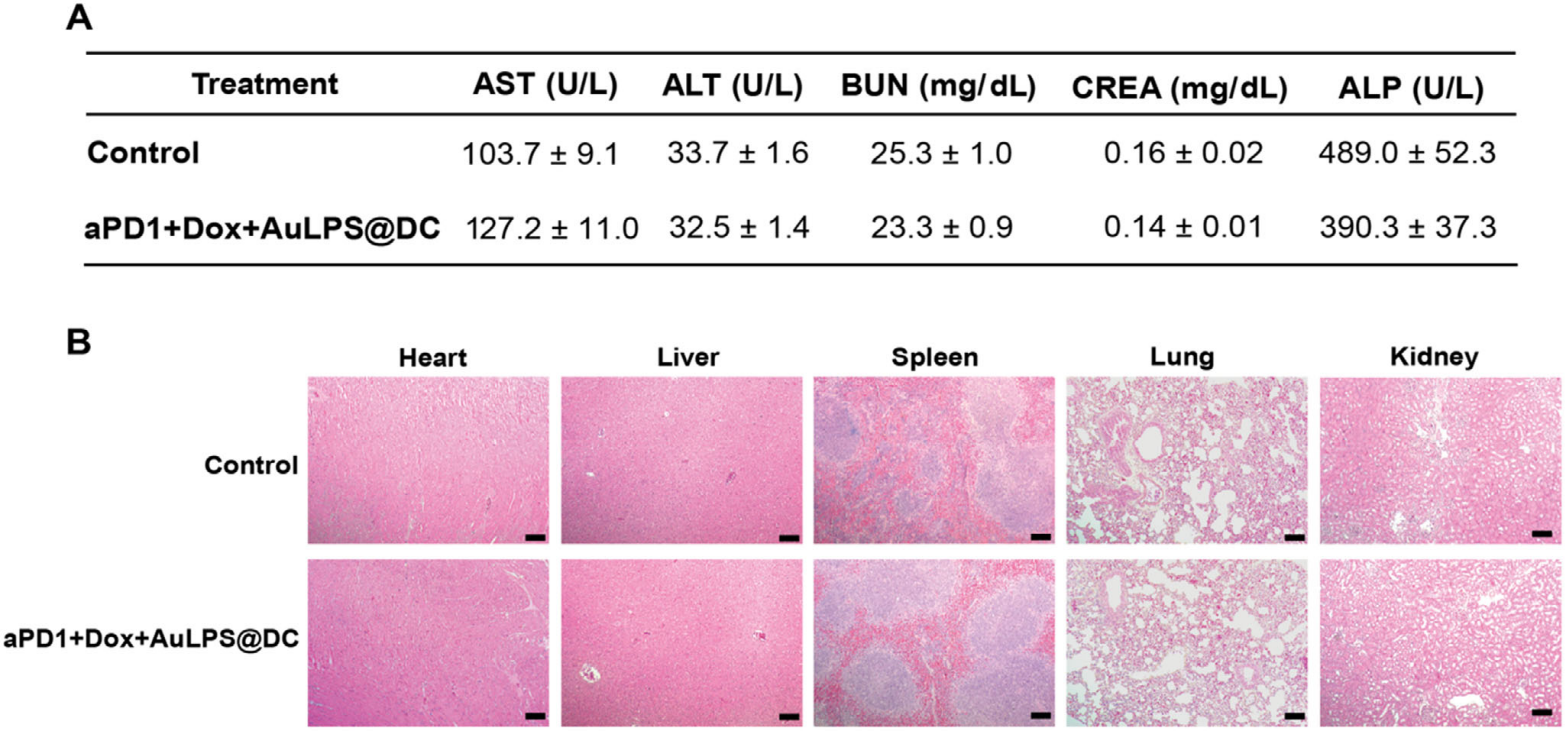
Toxicity profile of aPD1+Dox+AuLPS@DC in cryogel. A) Serum was collected from healthy BALB/c mice 24 h after subcutaneous injection of aPD1+Dox+AuLPS@DC in cryogel to evaluate hepatic enzyme levels and renal function markers. Abbreviations: ALT, alanine aminotransferase; AST, aspartate aminotransferase; ALP, alkaline phosphatase; BUN, blood urea nitrogen; CREA, creatinine (*n* = 7). Data are shown as the mean values ± SEM. B) Evaluation of systemic toxicity by H&E staining showing no histopathological changes in the major organs 24 h after subcutaneous injections of aPD1+Dox+AuLPS@DC in cryogel. Scale bar = 100 µm.

To assess the in vivo safety of aPD‐1+Dox+AuLPS@DC in cryogel, the levels of serum aspartate transaminase (AST), alanine transaminase (ALT), alkaline phosphatase (ALP), creatinine (CREA), and blood urea nitrogen (BUN) were examined in BALB/c mice treated with the formulation. Compared to the healthy control group, no significant changes in hepatic enzyme levels [AST, ALT, and ALP] or renal function indices (BUN and CREA) were observed following aPD‐1+Dox+AuLPS@DC in cryogel treatment (**Figure** **7**A). Evaluation of systemic toxicities by H&E staining showed no histopathological changes in the major visceral organs after treatment with aPD‐1+Dox+AuLPS@DC in cryogel (Figure 7B), providing clear evidence of the absence of significant in vivo toxicity.

## 3 Conclusion

This study highlights the potential of cryogel‐based DC immunotherapy incorporating AuLPS‐loaded DCs (AuLPS@DC), Dox, and an anti‐PD‐1 antibody as an effective post‐surgical strategy for TNBC. Following implantation at the surgical site, Dox induces ICD, releasing tumor antigens that are captured by AuLPS@DCs within the cryogel, enabling local antigen sensing and in situ DC activation. These DCs mature and migrate to TDLNs via the CCR7/CCL21 axis, promoting a robust expansion of CD8⁺ cytotoxic T cells. Incorporation of PD‐1 blockade further enhances T cell activation and supports durable antitumor immunity. A single administration of aPD‐1+Dox+AuLPS@DC cryogel significantly delayed tumor recurrence, suppressed lung metastasis, and prolonged survival compared to other treatment groups. The sustained local release of immune‐modulating agents minimized systemic toxicity. Overall, this triple‐component cryogel platform offers a clinically translatable post‐surgical immunotherapy capable of sustained immune activation and long‐term tumor control, representing a promising approach for improving TNBC outcomes.

## 4 Experimental Section

### Additional Materials and Methods

Additional materials and methods were included in the online Supporting Information (see online Supplemental Materials and Methods).

### Cells and Materials

The murine cell line 4T1 was purchased from the Bioresource Collection and Research Center (BCRC, Hsinchu, Taiwan) and cultured in high‐glucose Dulbecco's modified Eagle's medium (DMEM, SH30022.01, HyClone, GE Healthcare Life Sciences, USA) containing 10% fetal bovine serum (FBS, SH30084.03, HyClone, GE Healthcare Life Sciences, USA) and 1% penicillin/streptomycin solution. The G4NH₂ dendrimer, HAuCl₄, and LPS (Escherichia coli 0111:B4) were obtained from Sigma, Inc. (San Diego, CA, USA).

### Preparation of AuLPS NPs

The AuLPS nanoparticles (AuLPS NPs) were prepared by co‐assembling hydrophilic gold nanodots (AuNDs–NH₂) with lipopolysaccharide (LPS) to form stable nanovesicles. To synthesize AuNDs–NH₂, 200 µL of 150 mm HAuCl₄ was added to 20 mL of deionized water containing G4NH₂ dendrimers. The mixture was incubated at 4 °C overnight and then subjected to microwave irradiation at 120 °C for 30 min using a CEM Discover LabMate system. The resulting gold nanodots were purified by filtration through a 3 kDa molecular weight cutoff (MWCO) membrane filter, and residual AuCl₄ was removed via anion exchange chromatography (Fractogel EMD TMAE Hicap, Merck). For AuLPS NP assembly, AuNDs–NH₂ (10 mg mL^−1^) were mixed with LPS (100 µg mL^−1^) in serum‐free medium, following a protocol adapted from a previous publication.^[^ ^26^ ^]^

### Synthesis of GelMA and GelMA Cryogel

GelMA was synthesized by dissolving type A porcine skin gelatin (Sigma) (10% w/v) in PBS with stirring at 50 °C for 1 h. Methacrylic anhydride (Sigma) was added dropwise at a final volume ratio of 1:4 (methacrylic anhydride:gelatin solution). The solution was stirred at 50 °C for 1 h and then diluted 5 × with PBS. The resulting mixture was dialyzed in 12–14 kDa molecular weight cutoff tubing (Spectrum Labs) for four days against distilled water (dH₂O), with frequent water replacement. The dialyzed solution was then lyophilized, and the resulting GelMA was stored at −20 °C until use.

Cryogels were formed by dissolving GelMA in dH₂O at 0.75% (w/v) in the presence of 0.5% (w/v) ammonium persulfate (APS; Sigma) and 0.1% (w/v) tetramethylethylenediamine (TEMED; Sigma). Next, 250 µL of the prepolymer solution was pipetted into a cylindrical glass mold (8 mm diameter) and placed in a freezer set at −20 °C. Cryopolymerization was allowed to proceed for 18 h, after which the resulting cryogels were thawed and hydrated in dH₂O before use.

For the preparation of Dox‐loaded cryogels, 25 µg of doxorubicin was added to 300 µL of 0.75% (w/v) GelMA solution prior to polymerization. After gelation, the cryogels were washed with 1 mL of PBS for 20 min to remove unencapsulated drug. To prepare Dox and AuLPS@DC‐loaded cryogels, 4 × 10⁶ BMDCs were cultured with AuLPS NPs in 10 mL of medium for 24 h. After incubation, 1 × 10⁶ AuLPS‐treated BMDCs were seeded onto the doxorubicin‐loaded cryogels and allowed to adhere for 2 h prior to implantation.

### Bone Marrow‐Derived Dendritic Cell Culture and Treatment

Primary bone marrow cells were isolated from femurs and tibias of 6‐ to 12‐week‐old BALB/cByJ (BALB/c) mice after euthanasia. The bones were washed in 10% ethanol and placed in a petri dish containing sterilized PBS. Both epiphyses were removed using sterilized scissors and forceps. Bone marrow was flushed out using a syringe filled with isolation medium (RPMI supplemented with 10% heat‐inactivated FBS, 1% P/S, and 50 µm β‐mercaptoethanol) into another petri dish. A plastic pipette was used to homogenize the bone marrow. The sample was centrifuged at 1500 rpm for 5 min, washed with PBS, and the pellet was gently resuspended in isolation medium before being filtered through a 70 µm filter. Bone marrow cells were then seeded at a density of 4 × 10⁶ cells per plate and differentiated into dendritic cells in the presence of GM‐CSF (20 ng mL^−1^) for seven days. Bone marrow‐derived dendritic cells (1.2 × 10⁶ cells) were seeded in a 12‐well plate and treated with different formulations for 24 h. RNA was then extracted for quantitative PCR.

For in vitro study of STING activation and DC maturation, tumor cells were cultured overnight in 12‐well plates at an initial seeding density of 2 × 10⁵ cells. The next day, they were treated for 12 h with culture media containing either a control substance or doxorubicin (Dox). After treatment, the supernatants from these tumor cells were collected and used to incubate bone marrow‐derived dendritic cells (BMDCs) for 24 h. BMDCs were initially seeded at a density of 5 × 10⁵ cells per well in 12‐well plates and pretreated with different formulations for 24 h before exposure to the tumor cell supernatants. After treatment, dendritic cells were harvested for flow cytometry, stained with specific antibodies, or lysed for subsequent Western blot analysis.

### Animals and Tumor Models

BALB/cByJ female mice were purchased from the National Laboratory Animal Center (Taipei, Taiwan). To establish tumor models, 4T1 cells (1 × 10⁵ cells in 40 µL PBS) were subcutaneously injected into the right fourth mammary gland of 5‐ to 6‐week‐old BALB/cByJ female mice. Two weeks after tumor inoculation, surgery was performed to remove the tumors, and the cryogel was immediately implanted at the tumor site.

To analyze post‐treatment changes in immune cell profiles in tumors and tumor‐draining lymph nodes (TDLNs), mice were sacrificed two weeks after cryogel implantation. To evaluate spontaneous pulmonary metastasis, lungs were harvested and analyzed on day 14 post‐surgery from mice treated with the various formulations. All animals received humane care in compliance with the *Guide for the Care and Use of Laboratory Animals* published by the National Academy of Sciences. All study procedures and protocols were approved by the Animal Research Committee of National Tsing Hua University (Hsinchu, Taiwan).

### Next‐Generation Sequencing Gene Expression Analysis

The integrity of RNA extracted from the treatment and control groups using the RNeasy Kit (Qiagen) was evaluated with the RNA Nano6000 assay kit (Agilent Technologies, CA, USA). Library preparation and sequencing were conducted by Biotools Co., Ltd. The output data (FASTQ files) were mapped to the target genome using TopHat v2.0.12. HTSeq v0.6.1 was then used to count the number of reads mapped to each gene. The FPKM of each gene was subsequently calculated based on the gene length and the read counts mapped to that gene.

Gene expression values were calculated using read counts obtained from the alignment analysis. For relative gene expression analysis, normalization and differential expression gene (DEG) analysis were performed using edgeR (v3.28.1) and DESeq2 (v1.26.0). After edgeR analysis, genes with a *p*‐value and false discovery rate (FDR) < 0.05 were considered significantly differentially expressed. The ClustVis free web server and GSEA were used to analyze differential expression heatmaps and biological variability, respectively. The data were submitted to and approved by the Gene Expression Omnibus (GEO) with accession number GSE289340.

### IL‐12 and IL‐10 Enzyme‐Linked Immunosorbent Assay (ELISA)

BMDCs (5 × 10⁵ cells per well; 12‐well plate) were treated with different formulations for 12 or 24 h. IL‐12 and IL‐10 concentrations in the medium were determined using a mouse IL‐12 or IL‐10 ELISA kit (R&D Systems, Inc.), following the manufacturer's instructions.

### Transwell Assay for BMDC Migration

The migration assay was performed using 24‐well Transwell plates containing 8‐µm pore‐size polycarbonate filters (Corning, Life Sciences). CCL21 (50 ng mL^−1^ in a total volume of 600 µL) was added to the lower chambers. Mature dendritic cells (mDCs) were added to the upper chamber (1 × 10⁵ cells in a total volume of 100 µL) and incubated for 12 h at 37 °C. The number of migrated DCs in the lower chamber was determined. The number of spontaneously migrated DCs in the absence of chemokine was subtracted as background.

## Conflict of Interest

The authors declare no conflict of interest.

## Supporting information



Supporting Information

## Data Availability

The data that support the findings of this study are available from the corresponding author upon reasonable request.
