# Supplementary material for: Cryogel‐Based Dendritic Cell Immunotherapy for Post‐Surgical Breast Cancer Treatment
Source: Adv Sci (Weinh). 2025 Jul 11;12(37):e03238. doi: 10.1002/advs.202503238 (PMC12499431; doi:10.1002/advs.202503238)

ADVANCED SCIENCE
Open Access

Supporting Information

for *Adv. Sci.*, DOI 10.1002/advs.202503238

Cryogel-Based Dendritic Cell Immunotherapy for Post-Surgical Breast Cancer Treatment

Lam-Duc-Huy Nguyen, Sheng-Liang Cheng, Yu-Ting Yen, Hsin-Mei Lee, Te-Haw Wu, Jane Wang, Shu-Yi Lin* and Yunching Chen**

Supporting Information

Cryogel-based dendritic cell immunotherapy for post-surgical breast cancer treatment

Lam-Duc-Huy Nguyen[1,2,#], Sheng-Liang Cheng[1,3 #], Yu-Ting Yen[4,#], Hsin-Mei Lee[1], Te-Haw Wu[5,6], Jane Wang[2], Shu-Yi Lin[5,6]*, Yunching Chen[1,6]**

Supplementary Method and Materials

Transmission Electron Microscopy

The mixtures of LPS and AuNDs-NH_2 were prepared in DI water. Samples were mounted on a 400-mesh Cu grid with a carbon support and stained with a 2% uranyl acetate solution. Excess uranyl acetate was removed using filter paper, and the grid was dried before transmission electron microscopy imaging. Measurements were performed using a Hitachi H-7650 (Japan) at 100 kV and a field emission gun transmission electron microscope at 200 keV (JEOL, JEM-2100F, Japan).

Flow Cytometry

To assess DC maturation *in vitro*, BMDCs (5×10^5 cells/well; 12-well plate) were treated with conditioned medium from Dox-treated 4T1 cells (Dox), AuLPS NPs, or a combination of both for 24 h. The cells were then detached and stained with the following antibodies: CD45-FITC (no. 30-F11), CD11c-APC (no. 550261), and CD86-PE-Cy7 (no. 560582) from BD Biosciences (California, USA); MHC II-APC-eFluor780 (no. 47-5321-82) from eBioscience (California, USA). DC activation was determined by analyzing the co-expression of CD86 and MHC II, markers associated with DC activation, within the $CD45^+/CD11c^+$ DC population.

To assess post-treatment changes in immune cell profiles, tumor-bearing mice were perfused via intracardiac injection of PBS. Tumor tissues, lymph nodes, and cryogels were harvested and minced. Tumor tissues and cryogels were further digested at 37°C for 1 h in RPMI containing collagenase type 1A (1.5 mg/mL) and hyaluronidase (1.5 mg/mL). Digestion mixtures were filtered through 40-μm cell strainers. Single-cell suspensions were incubated with immunostained antibodies, washed, and resuspended in cold buffer (1% BSA in PBS). A 7AAD reagent (eBioscience) was added to the stained tubes (5 μL/tube) immediately before flow cytometry analysis. Flow cytometry data were acquired on an LSRII flow cytometer (Becton Dickinson) and

analyzed using FACSDiva™ software. The gating strategy for flow cytometry analysis can be found in **Figure S9**.

The following monoclonal anti-mouse antibodies were used: CD4-PE (no. RM4-5), CD3-APC (no. 145-2C11), CD8-PE-Cy7 (no. 53-6.7), CD45-FITC (no. 30-F11), CD11c-APC (no. 550261), CD86-PE-Cy7 (no. 560582), MHC II-PE, Granzyme B-FITC (no. 561998), and IFNγ-APC-Cy7 (no. 561479) from BD Biosciences (California, USA); MHC II-APC-eFluor780 (no. 47-5321-82) from eBioscience (California, USA).

Real-Time PCR

RNA was extracted using TRIzol™ Reagent (Invitrogen, 15596018, USA) according to the manufacturer's instructions. Total RNA was then reverse-transcribed into cDNA using the High-Capacity cDNA Reverse Transcription Kit (Applied Biosystems, USA) in a SimpliAmp Thermal Cycler (Thermo Fisher Scientific, MA, USA), following the manufacturer's instructions.

Quantitative PCR was performed using SYBR Green Real-Time PCR Master Mixes (Applied Biosystems, USA) on a QuantStudio 3 Real-Time PCR System (Applied Biosystems, USA). Gene expression levels were evaluated using the Ct method in all tested samples and normalized to β-actin as an internal control. The primer sequences are listed as follows:

Primer	Forward sequence (5'-3')	Reverse sequence (3'-5')
mβ-actin	TGAGAGGGAAATCGTGCGTG	TTGCTGATCCACATCTGCTGG
mIL-6	TGATGCACTTGCAGAAAACA	ACCAGAGGAAATTTTCAATAGGC
mIL-10	CCAGAGCCACATGCTCCTA	AGGGGAGAAATCGATGACAG
mIL-33	AGGCGACGGTGTGGATGGGA	CGTCACCCCTTTGAAGCTCCACG
mIL-12α	GCCAGGTGTCTTAGCCAGTC	AGCTCCCTCTTGTTGTGGAA
mCXCL1	TCCAGAGCTTGAAGGTGTTGCC	AACCAAGGGAGCTTCAGGGTCA
mCXCL2	CTCCTTTCCAGGTCAGTTAGC	CAGAAGTCATAGCCACTCTCAA
mCXCL9	AGTGTGGAGTTCGAGGAACC	GAGTCCGGATCTAGGCAGG

mCXCL10	CCAAGTGCTGCCGTCATTTTC	GGCTCGCAGGGATGATTTCAA
mCXCL11	AGCTGCTCAAGGCTTCCTTA	AGTAACAATCACTTCAACTTTGTCG
mCCL5	TGCCCACGTCAAGGAGTATTT	TCGAGTGACAAACACGACTGC
mCCL17	TGCTTCTGGGGACTTTTCTG	TGGCCTTCTTCACATGTTTG
mCCL22	GTCCTTCTTGCTGTGGCAAT	ACGGTTATCAAAACAACGCC
mIFNα	TGTCTGATGCAGCAGGTGG	AAGACAGGGCTCTCCAGAC
mIFNβ	ATGGTGGTCCGAGCAGAGAT	CCACCACTCATTCTGAGGCA
mIFNγ	CAGCAACAGCAAGGCGAAAAAGG	TTTCCGCTTCCTGAGGCTGGAT
mCCR7	AGAGGCTCAAGACCATGACGGA	TCCAGGACTTGGCTTCGCTGTA

Western Blot Analysis

BMDCs (5×10^5 cells/well; 12-well plate) were treated with conditioned medium from Dox-treated 4T1 cells (Dox), AuLPS NPs, or a combination of both for 24 h. The cells were then lysed using RIPA buffer on ice for 10 min. The lysed cell solution was mixed with 4× Laemmli sample buffer (Bio-Rad, California, USA) and subsequently heated at 95°C. The cell lysates were separated on a 10% acrylamide gel and transferred onto a PVDF membrane. The membranes were blocked for 1 h with 5% bovine serum albumin (BSA) in TBST and then incubated overnight at 4°C with primary antibodies against phospho-TBK1 (Ser172) (D52C2) (no. 5483, Cell Signaling Technologies, Massachusetts, USA), TBK1 (D1B4) (no. 3504, Cell Signaling Technologies, Massachusetts, USA), phospho-IRF3 (Ser396) (D6O1M) (no. 29047, Cell Signaling Technologies, Massachusetts, USA), IRF3 (D83B9) (no. 4303, Cell Signaling Technologies), and β-actin (no. A5441, Sigma Aldrich, Missouri, USA).

Figure S1. Cytokine secretion from BMDCs was measured by ELISA 24 h after treatment with free LPS, Au NPs or AuLPS NPs (n=3). All data are shown as the mean ± SEM. ****P*<0.001.

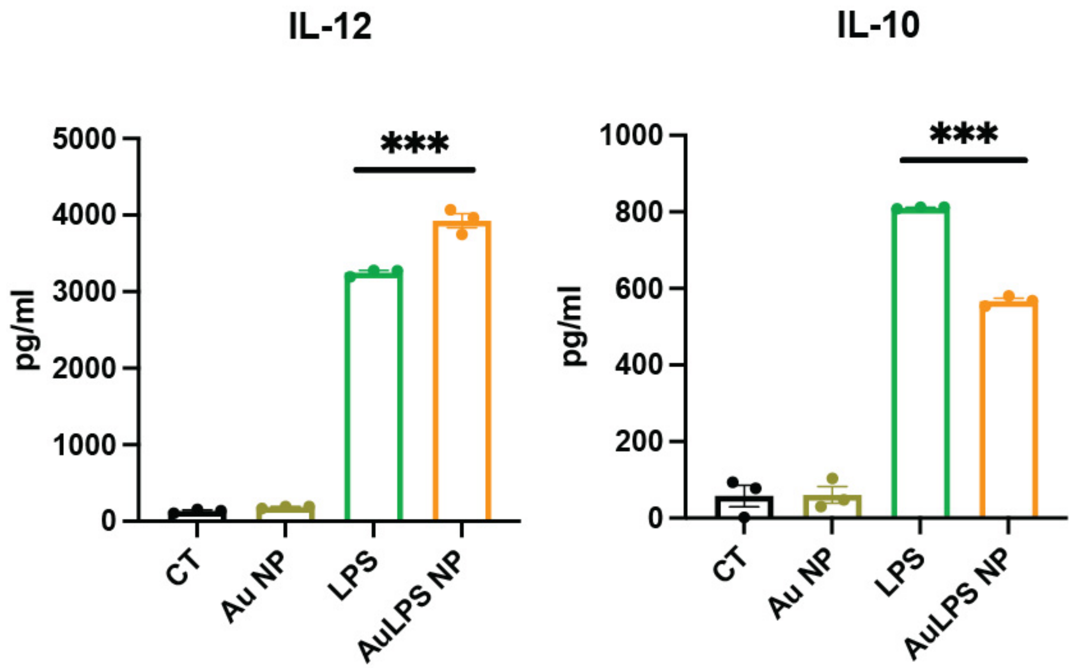

Figure S2. IL-12 secretion from BMDCs was measured by ELISA 24 hours after treatment with conditioned medium from Dox-treated 4T1 cells (Dox), AuLPS NPs, or their combination (n = 3). Data are presented as mean ± SEM. *P < 0.05, ***P < 0.001.

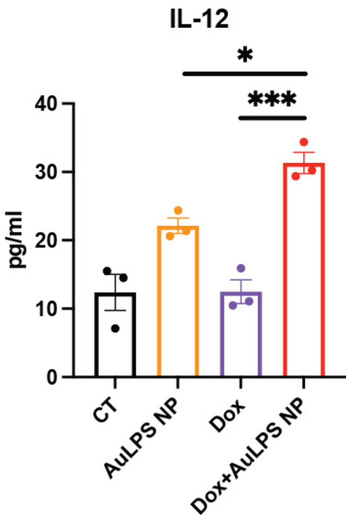

Figure S3. Flow cytometry analysis of MHC II and CD86 expression in BMDCs treated with conditioned medium from Dox-treated 4T1 cells (Dox), AuLPS NPs, or a combination of both for 24 h. Representative FACS plot of activated BMDCs ($CD86^{+}MHCII^{+}$) (A), and representative flow histogram plots of CD86 (B) and MHCII (C).

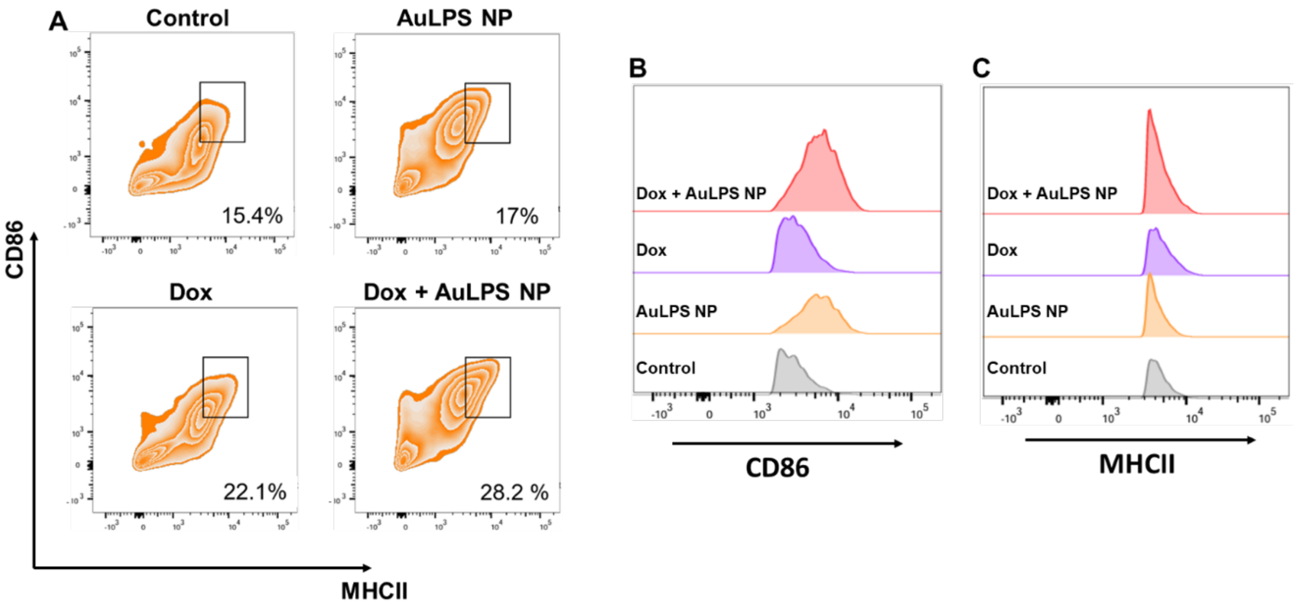

Figure S4. BMDC seeding efficiency. (n=4) Data are presented as mean ± SEM. *P < 0.05.

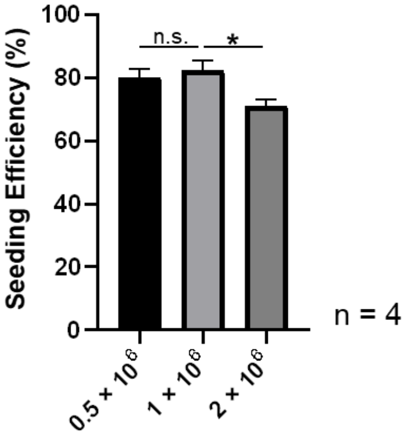

Figure S5. Upregulation of CCR7 Expression in BMDCs Treated with AuLPS NPs. Flow cytometry analysis of CCR7 expression in BMDCs treated with AuLPS NPs for 24 hours (n = 4). All data are presented as the mean ± SEM. **P < 0.01.

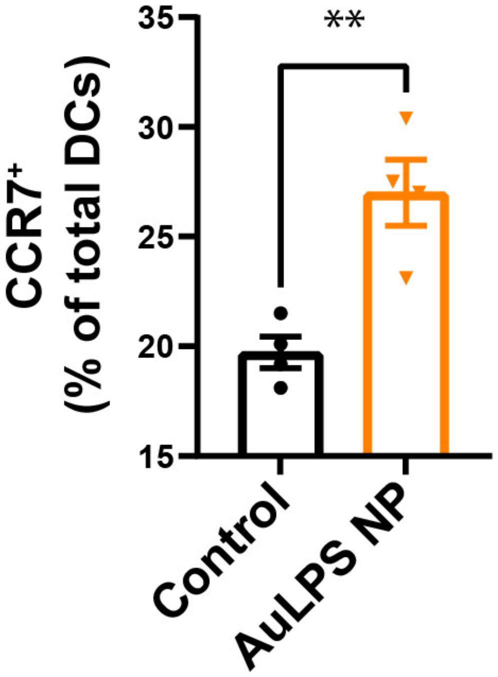

Figure S6. (A) Schematic of the experimental protocol. On day -14, 1 × 10^5 4T1 cells were injected into the mammary gland of BALB/c mice. The implanted tumor was surgically removed on day 0, and various formulations were implanted into the surgical site (BMDC: 1 × 10^6 cells/mouse). Cryogel, tumors and TDLNs were analyzed on day 2. MHC II and CD86 expression in delivered BMDCs in cryogel (B) tumor (C) or TDLNs (D) 48 h after treatment with BMDCs alone, cryogels containing BMDCs or AuLPS@DCs were analyzed by flow cytometry (n = 4). All data are shown as the mean ± SEM. *P<0.05, **P<0.01.

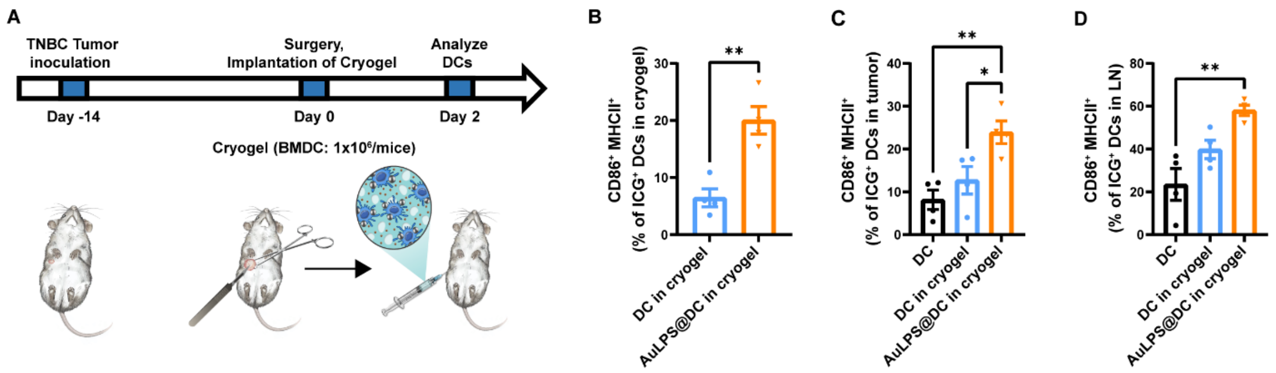

Figure S7. The cryogel containing AuLPS@DC and Dox induces potent antitumor immunity. Representative FACS plots showing: (A) DCs ($CD45^+CD11c^+$) in tumors; (B) activated DCs ($CD86^+MHCII^+$) in tumors; (C) activated DCs in tumor-draining lymph nodes (TDLNs); (D) cytotoxic $CD8^+$ T lymphocytes ($CD3^+CD8^+$) in tumors; (E) IFN-γ^+ $CD8^+$ T cells in tumors; (F) IFN-γ^+ $CD8^+$ T cells in TDLNs; (G) granzyme B^+ $CD8^+$ T cells in tumors; and (H) granzyme B^+ $CD8^+$ T cells in TDLNs.

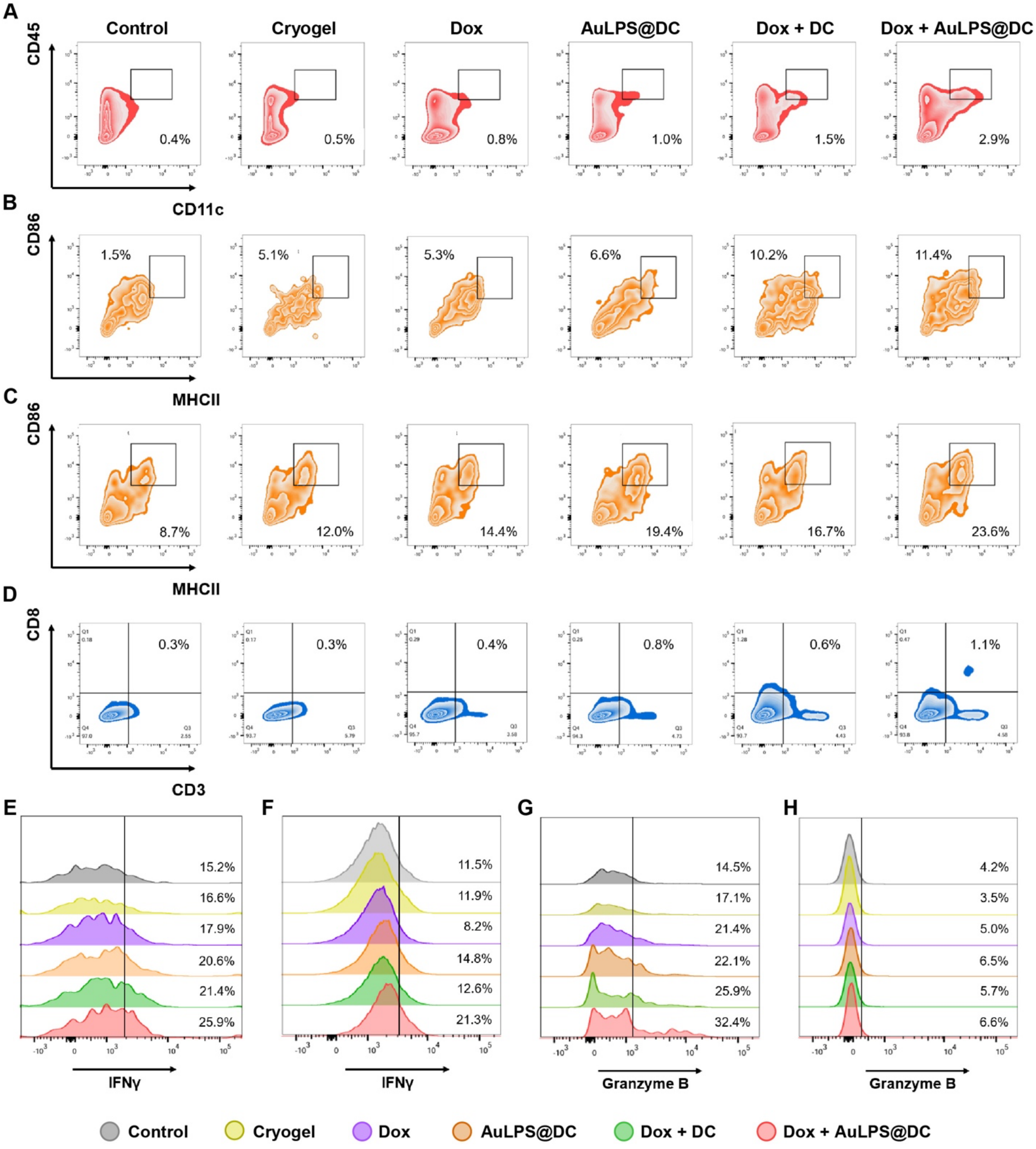

Figure S8: T helper cells ($CD3^{+}CD4^{+}$ T cells) in tumor (A) and tumor draining lymph nodes (B) of mice were detected by flow cytometry (n=8). All data are shown as the mean ± SEM

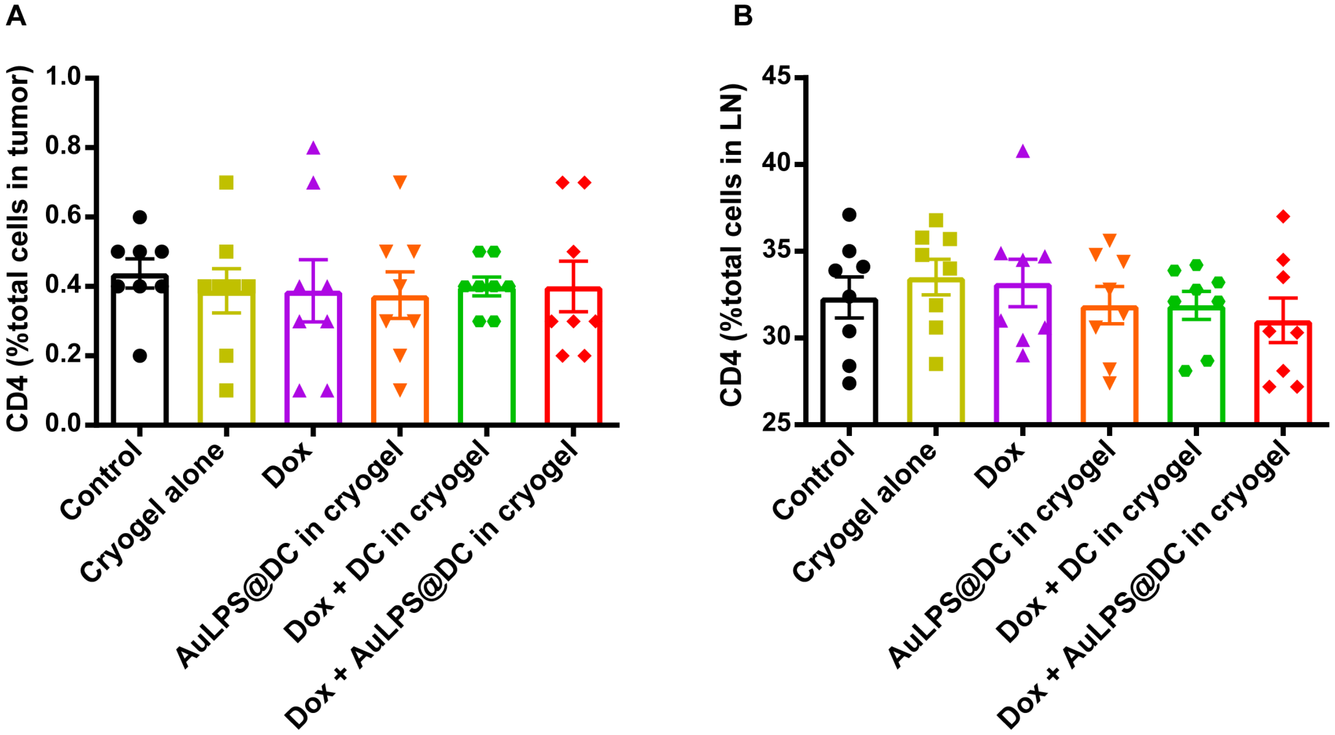

Figure S9: Gating strategy for flow cytometry to determine T cell (A) and DC subsets (B) in lymph nodes

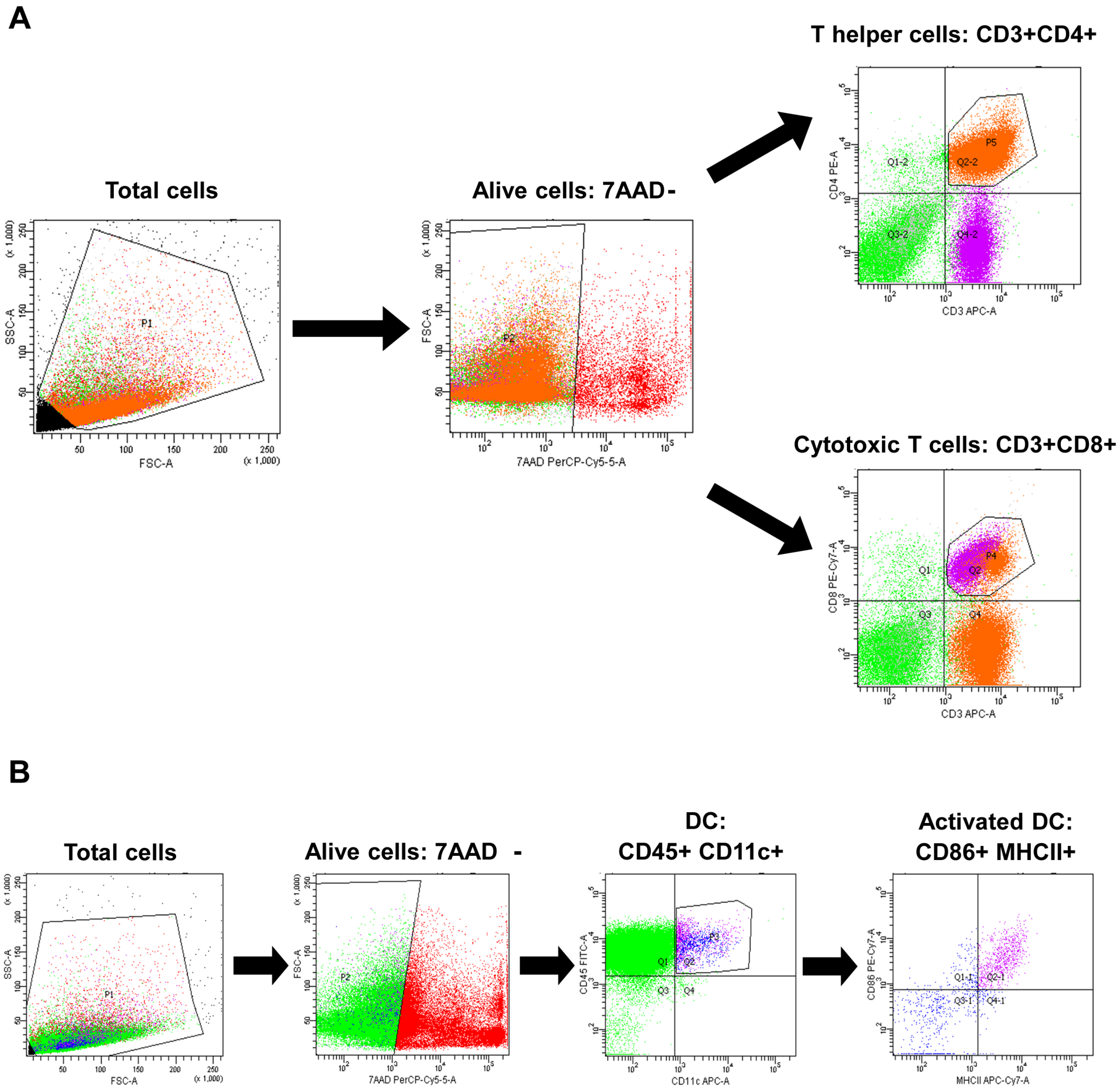

Supplement: Supplementary file 1 — Supporting Information [file ADVS-12-e03238-s001.pdf]
